# Response to: Comment on “Neutrophil-to-Lymphocyte Ratio and Platelet-to-Lymphocyte Ratio: Novel Markers for Diagnosis and Prognosis in Patients with Idiopathic Sudden Sensorineural Hearing Loss”

**DOI:** 10.1155/2015/583738

**Published:** 2015-05-18

**Authors:** Young Joon Seo, Junhui Jeong, Jae Young Choi, In Seok Moon

**Affiliations:** ^1^Department of Otorhinolaryngology, Yonsei University Wonju College of Medicine, Wonju, Republic of Korea; ^2^Department of Otorhinolaryngology, Yonsei University College of Medicine, Seoul, Republic of Korea

We are very pleased to get those comments on our paper, because those things were important considerations we thought in these results. But we have to comment against those questions.

1. The WBC counts could reflect an acute inflammation [1]. We excluded patients not only by WBC counts. We excluded the patients with fever, physical exam, and medical history (because the WBC count was performed at the initial visit, we could discriminate acute inflammations with physical exams). We excluded patients over 11000/u (because 10000 is upper limit in our hospital, but 10% error could be possible) [2]. So we add 1000/u to the normal value. We think it could be acceptable universally. And the findings of the increasing in the WBC and neutrophil are important findings, though they had no evidence of acute inflammations. The author misunderstood the subject of this study.
2. DM is so important risk factor for sudden SNHL. Glucose levels affect vascular insufficiency to make sudden SNHL. We did already consider that in the discussion. This study was retrospectively performed. We could not control the case group. So we mentioned these limitations in the discussion [3, 4]. “Although glucose between the patients group and the control group was significantly different, because we could not control the sugar test with fasting glucose test for all patients and control group, we cannot be sure the value as the important risk factor.”
3. Before binary logistic regression analysis, univariate logistic regression analysis is important. But WBC includes neutrophil, lymphocyte, and monocyte [5]. Changes of neutrophil automatically affect lymphocyte. We did univariate logistic regression analysis, but in this statistics, binary logistic regression would be preferred.
4. And we did not want to point out the cut-off value. We need to study more about that in the future. In conclusion, the author considered so important points. But they did not consider this study was a retrospective study and we already thought about those things. So we want to reconsider for the publication.
